# When Treatment Turns to Stone: Injection‐Site Calcinosis Associated With Interferon‐β in Multiple Sclerosis: A Case Report

**DOI:** 10.1002/ccr3.72931

**Published:** 2026-06-14

**Authors:** Anna Walter, Adam Bott, Marianne Kiechle, Margret Hund‐Georgiadis

**Affiliations:** ^1^ REHAB Basel, Clinic for Neurorehabilitation and Paraplegiology Basel Switzerland

**Keywords:** adverse effect, case report, injection‐site calcinosis, interferon beta, multiple sclerosis

## Abstract

Injection‐site calcinosis is a rare complication of interferon‐β therapy in multiple sclerosis. We report a 52‐year‐old woman with stable bilateral gluteal calcifications after long‐term subcutaneous interferon‐β‐1b therapy. Findings were most consistent with probable dystrophic calcinosis cutis secondary to chronic repeated injection‐site trauma.

## Key Clinical Message

Injection‐site calcinosis is a rare but clinically relevant complication of long‐term interferon therapy that should be considered in patients with persistent or atypical local reactions. Awareness of this entity may help avoid misdiagnosis, unnecessary investigations, and delayed treatment modification.

## 1 Introduction

Multiple sclerosis is a chronic inflammatory demyelinating disease of the central nervous system and a leading cause of neurological disability in young and middle‐aged adults [1]. Interferon‐β preparations have been established therapies for relapsing forms of MS for more than three decades [2, 3].

Cutaneous adverse reactions such as erythema, induration, lipoatrophy, and necrosis are well described during interferon therapy [4, 5]. Severe injection‐site reactions may persist for weeks to months and can result in permanent tissue changes [4]. Persistent calcified lesions, however, are rare. We report a case of probable localized injection‐site dystrophic calcinosis cutis associated with long‐term repeated subcutaneous interferon‐β‐1b injections.

## 2 Case History

A 52‐year‐old Caucasian woman was admitted to inpatient rehabilitation following hip arthroplasty due to a left femoral fracture. At admission, she had an Expanded Disability Status Scale score of 4 due to ataxia related to long‐standing multiple sclerosis. The disease was diagnosed in 2001 and progressed to secondary progressive MS in 2018.

Initial immunomodulatory therapy began in 2003 with subcutaneous interferon‐β‐1b administered every other day until 2008. Subsequent disease‐modifying therapies included natalizumab (2009–2012), fingolimod (2012–2018), siponimod (2021–2023), and currently ocrelizumab administered biannually. Among these therapies, only interferon‐β‐1b had been administered subcutaneously on a regular basis.

**FIGURE 1:**
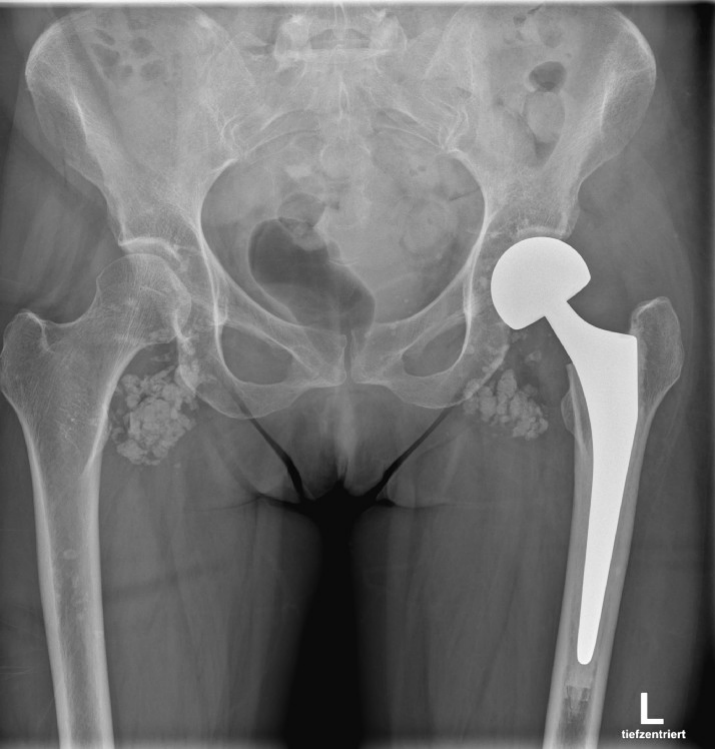
Anteroposterior pelvic radiograph demonstrating multiple bilateral clustered calcified subcutaneous lesions within the gluteal regions adjacent to the ischiopubic rami. The calcifications were identified incidentally during post‐operative imaging following left hip arthroplasty.

Clinical examination revealed numerous firm, non‐tender, indurated subcutaneous nodules in the bilateral gluteal folds. Although the patient had long been aware of these lesions without significant discomfort or functional impairment, the calcifications were identified incidentally on routine post‐operative pelvic radiographs performed during rehabilitation after hip arthroplasty. Repeated physical examinations revealed no additional skin abnormalities. Pelvic radiographs demonstrated multiple well‐defined bilateral calcifications (Figure 1). To assess temporal stability, prior radiographs dating back to 2015 were reviewed and demonstrated unchanged lesions in both size and number, indicating long‐term stability. No additional imaging beyond radiographs was performed.

The patient reported repeatedly injecting interferon‐β‐1b into these areas because they were perceived as less painful. No other medications had been administered subcutaneously in these areas.

## 3 Differential Diagnosis, Investigations and Treatment

Laboratory investigations, including serum calcium and phosphate levels, were within normal limits, arguing against metabolic causes of calcification. Differential diagnoses included heterotopic ossification, metastatic calcification, injection‐related granulomatous reactions, infectious processes, and dystrophic calcinosis cutis [6, 7].

**FIGURE 2:**
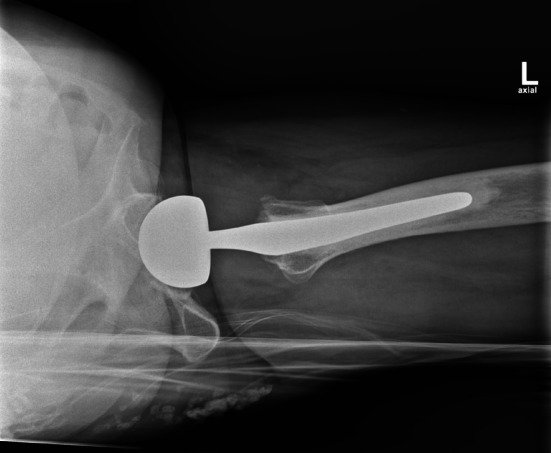
Axial radiographic view of the left hip demonstrating lobulated calcified subcutaneous deposits within the gluteal soft tissues, consistent with probable localized dystrophic calcinosis cutis at chronic interferon‐β‐1b injection sites.

Given the characteristic localization at repeated injection sites, normal laboratory findings, chronic radiographic stability, and absence of systemic abnormalities, the findings were considered most consistent with probable localized dystrophic (iatrogenic) calcinosis cutis secondary to long‐term repeated mechanical tissue trauma from subcutaneous injections (Figure 2). Histopathological confirmation was not pursued because the patient remained asymptomatic and imaging findings were stable over time.

Conservative management and patient education regarding systematic injection‐site rotation were recommended. Written informed consent for publication was obtained from the patient.

## 4 Conclusion and Results (Outcome and Follow‐Up)

The calcified lesions remained clinically and radiographically stable during follow‐up without progression or need for intervention, and the patient remained asymptomatic.

Injection‐site calcinosis should be considered in patients with persistent or atypical local reactions during or after long‐term interferon therapy. Recognition of this rare complication is clinically relevant because persistent calcified lesions may mimic alternative pathological processes and potentially lead to unnecessary investigations or inappropriate management. In the present case, the findings were most likely related to chronic repeated mechanical tissue trauma rather than direct pharmacologic toxicity of interferon‐β.

## 5 Discussion

Injection‐site reactions are frequent during interferon therapy and may negatively affect adherence [8]. Most reactions are inflammatory or immune‐mediated, including hypersensitivity and local necrosis [4]. Only a very limited number of cases of injection‐associated calcinosis cutis have been reported in the literature, including reports related to subcutaneous therapies such as glatiramer acetate and calcium‐containing heparin [9, 10]. To our knowledge, reports specifically describing interferon‐associated injection‐site calcinosis remain exceptionally rare [4, 10].

Calcinosis cutis represents deposition of calcium salts in the skin and is classified as dystrophic, metastatic, idiopathic, or iatrogenic [6]. Dystrophic calcification occurs in previously damaged tissue despite normal serum calcium levels and may result from repeated local tissue injury, including repeated injections [6].

Because interferon‐β contains neither calcium nor phosphate, a direct pharmacologic mechanism appears unlikely. Instead, repetitive local tissue injury from long‐term subcutaneous injections is the most plausible explanation for the observed calcifications. This interpretation is further supported by the exclusive localization to chronic injection sites, normal metabolic investigations, and long‐term radiographic stability.

Preventive measures include systematic rotation of injection sites, atraumatic technique, and patient education [5].

In our patient, no evidence of autoimmune disease, infectious processes, or metabolic abnormalities was identified, further supporting a localized dystrophic process related to repeated injections.

This case highlights that persistent injection‐site abnormalities should not be attributed solely to common inflammatory reactions. Failure to recognize calcinosis cutis may result in unnecessary diagnostic procedures or inappropriate management, underscoring the importance of clinical awareness.

## 6 Limitations

This report describes a single patient and lacks histopathological confirmation. Therefore, definitive causality cannot be established, and the diagnosis remains probable rather than biopsy‐proven.

## Author Contributions

**Anna Walter:** conceptualization, investigation, writing – original draft, project administration. **Marianne Kiechle:** conceptualization, investigation. **Adam Bott:** writing – review and editing, formal analysis, project administration. **Margret Hund‐Georgiadis:** supervision.

## Funding

The authors have nothing to report.

## Ethics Statement

Written informed consent was obtained from the patient for publication of this case report and accompanying images.

## Conflicts of Interest

The authors declare no conflicts of interest.

## Data Availability

Data sharing is not applicable to this article as no datasets were generated or analyzed beyond the clinical information described.
